# 4526 Coping with End of Life Care: Compassion Fatigue and Burnout Effects on Cortisol Patterns of Health Care Providers Caring for Children

**DOI:** 10.1017/cts.2020.232

**Published:** 2020-07-29

**Authors:** Liza Carolina Sanchez-Plazas, Ricardo L. Garcia, Kelly Komatz

**Affiliations:** 1 University of Puerto Rico, Medical Science Campus; 2 University of Florida, Jacksonville

## Abstract

OBJECTIVES/GOALS: The objectives are to assess the impact of cumulative grief on the development of Compassion Fatigue (CF) and Burnout Syndrome (BS) in HCPs who care for dying children. We will also evaluate the relationship between CF and cortisol patterns in HCPs. METHODS/STUDY POPULATION: Cross-sectional study to be conducted in a Pediatric Hospital in Puerto Rico. A sample of 50 pediatric nurses will be selected to collect the data to evaluate the occurrence of CF and BS among HCP caring for children during end of life (EoL). Study subjects will include nurses who care for dying children in the Intensive Care Units and Oncology ward. Nurses working in the pediatric ward will be included as the control group. Three validated instruments (Spanish Version) will be administered (Professional Quality of Life vIV, Maslach Burnout Inventory- HSS, briefCOPE scale). Cortisol samples in saliva and hair will also be taken to determine levels in these HCPs. RESULTS/ANTICIPATED RESULTS: Our expected outcome is that CF and BS will be more frequent in HCPs caring for children during EoL compared with controls and that EoL nurses will have higher scores on CF scale and more frequent dysregulated cortisol patterns. DISCUSSION/SIGNIFICANCE OF IMPACT: Understanding how HCPs cope with grief caused by child death and the occurrence of CF and BO in our hispanic population allowing us to develop support strategies based on the specific HCPs needs. This knowledge will help improve HCPs’ well-being and may diminish the physiologic impact on cortisol.

